# Exploring the Usability and Quality of a Mobile App–Based Intervention for Personalized Exercises in Individuals With Spinal Cord Injury: Convergent Parallel Mixed Methods Study

**DOI:** 10.2196/84316

**Published:** 2026-09-25

**Authors:** Sara Nataletti, Anushua Banerjee, Sara Prokup, Arun Jayaraman, Alex W K Wong

**Affiliations:** 1 Feinberg School of Medicine, Northwestern University, Chicago, IL, United States; 2 Shirley Ryan AbilityLab, 355 E. Erie Street, Chicago, IL, 60611, United States, 1 3122381742

**Keywords:** spinal cord injury, usability testing, mobile health, smartphone, exercise, adherence, mixed methods approach

## Abstract

**Background:**

Mobile health technologies offer scalable opportunities to support home-based exercise programs. However, most commercially available apps are not tailored to individuals with spinal cord injury (SCI) and have rarely been evaluated for usability and implementation outcomes in this population.

**Objective:**

This study aims to assess the acceptability, appropriateness, feasibility, and quality of a mobile app–based intervention designed to support exercise adherence in individuals with SCI.

**Methods:**

We conducted a convergent parallel mixed methods study using quantitative data from standardized measures and qualitative feedback from focus groups. Twelve individuals with SCI (mean age 50.5, SD 17 years; n=6, 50% male; n=8, 67% White; n=6, 50% with thoracic injuries; n=6, 50% ambulators) engaged in a customized exercise regimen using the app for 2 weeks. Acceptability, appropriateness, and feasibility of this mobile intervention were assessed using the Acceptability of Intervention Measure, Intervention Appropriateness Measure, and Feasibility of Intervention Measure. The objective and subjective qualities, and the perceived impact, of the app-based intervention were evaluated using the User Version of the Mobile Application Rating Scale.

**Results:**

Using a cutoff score of >4 out of 5, 67% (8/12) of participants rated the app-based intervention as highly acceptable and feasible, and 50% (6/12) rated it as highly appropriate. The objective quality of the app was rated 3.57 out of 5 on the User Version of the Mobile Application Rating Scale. At the scale level, information received the highest score (mean 4.10, SD 0.52), with functionality (mean 3.51, SD 0.66) and aesthetics (mean 3.50, SD 0.70) closely following. Engagement scored lowest (mean 3.18, SD 0.72). The app’s subjective quality averaged 3.27 (SD 0.88), and its perceived impact on exercise adherence was rated 3.79 (SD 0.92). Qualitative feedback indicated that customization, access to professional support, and SCI-specific exercises were positive features. Areas for improvement included bug fixes and enhanced features to boost engagement.

**Conclusions:**

The smartphone-based intervention demonstrates adequate acceptability and feasibility, along with high information quality and favorable usability, for supporting exercise adherence in individuals with SCI. Improvements in engagement features are recommended to enhance sustainable implementation.

## Introduction

The health benefits of physical activity are well established, yet many individuals with spinal cord injury (SCI) engage in lower levels of physical activity compared to the general population [1-5]. Low physical activity levels increase the risk of secondary health conditions, such as cardiovascular disease [6,7], diabetes [8], and obesity [9] and contribute to deconditioning [1,10,11], leaving many individuals unable to perform daily tasks independently.

Although international guidelines recommend regular aerobic and strength training exercise for people with SCI, adherence remains low [12]. Transportation difficulties, inaccessible fitness facilities, a shortage of trained professionals, financial constraints, and individual-level challenges such as low motivation, fear of injury, and uncertainty about appropriate exercises are common barriers to exercise experienced by people with SCI. Traditional exercise programs are often not well equipped to meet the needs of individuals with varying functional abilities, resulting in low participation and high dropout rates [5,13,14].

Home-based exercise programs can reduce some of these barriers by improving convenience and accessibility. However, home-based programs often require users to self-manage their exercise routine and typically lack real-time feedback or progression support from clinicians [15,16], making long-term adherence challenging.

Mobile health (mHealth) technologies, particularly smartphone-based apps, provide scalable and low-cost solutions to support home-based exercise interventions. Smartphones are widely adopted across income levels [17] and feature embedded sensors and communication tools that enable personalized feedback, remote monitoring, and adaptive intervention delivery [18,19]. Although a growing number of mHealth apps have been developed to support self-management and physical activity among individuals with SCI, existing tools still have notable limitations. Reviews of available apps have found that most focus primarily on general health monitoring or on the self-management of secondary conditions (eg, bowel, bladder, and pain management), rather than on supporting exercise adherence [20,21]. Among the few apps developed specifically for home-based exercise in SCI, one pilot study evaluated a smartphone app that delivered clinician-prescribed upper-limb exercises and included a gamified adherence system [22]. While promising, this approach was limited to upper-limb exercises within a single patient group and lacked real-time clinician interaction, ecological momentary assessment (EMA), and adaptive content delivery tailored to individual progress. Overall, most commercially available exercise apps are not tailored to the needs of individuals with SCI and often lack critical accessibility features, SCI-specific exercise content, or features for clinician interaction [20,23]. Despite generally adequate usability ratings, challenges related to long-term engagement and sustained adherence remain largely unaddressed [20]. Few interventions have been codeveloped with SCI users or formally evaluated in this population, particularly for usability and implementation [24].

To address this gap, we conducted an mHealth development study involving individuals with SCI, caregivers, and clinicians, working in conjunction with the app developers, to coadapt a smartphone-based app for home-based exercises in individuals with SCI. Feedback from this process informed the integration of SCI-specific exercise content, accessibility features, EMA of exertion and symptoms, in-app messaging with a clinician, motivational messages, and progress tracking. The app developers then customized the app accordingly, making it ready for a first formal usability evaluation among individuals with SCI [25].

Before widespread adoption, it is important to assess the app’s usability and quality related to the goals of the study. Evaluating key implementation outcomes, such as acceptability, appropriateness, and feasibility, helps determine whether an intervention is likely to be adopted and maintained in practice. Therefore, this study aimed to conduct a formative usability evaluation of the customized app using a mixed methods design. We evaluated quantitative implementation outcomes and gathered qualitative feedback through focus groups, capturing participants’ experiences with existing features and their suggestions for future enhancements. The findings will guide further refinement of specific app features by the app developers and inform the subsequent evaluation of this mHealth-based exercise intervention in a proof-of-concept efficacy study among individuals with SCI.

## Methods

### Study Design

This study adopted a convergent parallel mixed methods design, in which we collected, analyzed, and converged both quantitative and qualitative data. This formative evaluation served a dual purpose: to assess the usability of the app-based intervention and its integrated features among individuals with SCI, and to capture user input to inform further refinements and customizations implemented in conjunction with the app developers specific to the study needs.

### Participants

Participants with SCI were recruited via an official study website [26] or locally from a rehabilitation hospital’s clinical registry between March and July 2024. Eligible participants were English-proficient adults aged 18 years or older who had been diagnosed with SCI at least 1 year prior. Participants had either an incomplete injury at the C3 level or below or complete paraplegia and were not currently meeting the recommended SCI exercise guidelines [27]. Exclusion criteria included severe cognitive or visual impairments, inability or unwillingness to use a smartphone, or inability to download the study app.

### Study Procedures

After the initial screening, eligible participants met with the research team to complete the informed consent process, provide demographic and medical history information, and undergo a physical evaluation with the study’s physical therapist. During this session, participants received hands-on training on the study app and were prescribed a customized exercise program by a licensed physical therapist, tailored to their physical capacity, injury level, and personal goals. Before app setup, each participant completed a brief clinical assessment (including muscle strength testing, range-of-motion evaluation, and spasticity assessment) and a goal-setting session with the physical therapist to identify personal exercise goals. These findings informed the selection of appropriate exercises from a library of video-guided routines, with frequency and intensity tailored to SCI-specific guidelines and each participant’s functional abilities. The personalized program was then uploaded directly to the participant’s app account. Throughout the trial, participants could communicate with their physical therapist via in-app messaging and leave comments on their exercise sessions. The program was adjusted as needed in response to feedback and progress. Over the following 2-week trial, participants were instructed to complete and log their personalized exercise program at least twice per week and answer 4 short EMA surveys within the app daily. The EMA surveys were designed to capture participants’ real-time experiences with exercise, including perceived barriers (eg, low motivation, lack of equipment) and facilitators (eg, social support, energy levels), as well as exertion and symptoms during or after exercise. In this formative study, the EMA component also examined how feasible it was for users to complete several daily surveys within the app while also testing the EMA system’s functionality, scheduling, and delivery in a real-world home setting before moving on to the full efficacy study. However, due to technical issues with the study app, some participants did not receive the daily EMA surveys during their trial period. No other technical problems were reported by participants throughout the 2-week trial.

### Quantitative Data Collection

At the end of the 2-week trial, participants completed 3 standardized implementation measures [28]: the Acceptability of Intervention Measure (AIM), Intervention Appropriateness Measure (IAM), and Feasibility of Intervention Measure (FIM), along with the User Version of the Mobile Application Rating Scale (uMARS) to evaluate app usability and perceived quality [29,30]. Surveys were administered electronically via the REDCap platform. Research staff were available to help participants with survey completion if necessary. Specifically, AIM, IAM, and FIM each have 4 items rated on a 5-point Likert scale, from 1 (“completely disagree”) to 5 (“completely agree”), which measure users’ perceptions of the app’s acceptability, appropriateness, and feasibility related to the study, respectively. The uMARS uses a 5-point scale (1=“inadequate” to 5=“excellent”) to evaluate the objective quality of the app across four domains: engagement, functionality, aesthetics, and information, as well as the subjective quality of the app. uMARS also includes items evaluating the perceived impact of the app on users’ knowledge, attitudes, intentions to change, and the likelihood of actual change in the target behavior (ie, adhering to exercise guidelines) specific to the study. We defined a score of 4 or higher as a benchmark for high acceptability, appropriateness, feasibility, and quality [28,30].

### Qualitative Data Collection

Qualitative data were gathered through three focus groups conducted between May and August 2024, following participants’ completion of the postuse surveys. These sessions aimed to contextualize the quantitative findings and further explore participants’ experiences and perceptions of the intervention. Each focus group included 2 to 6 participants, lasted between 45 and 90 minutes, and was moderated by trained research staff. We adopted the focus group guide built from our prior development study [25] with questions guided by the Consolidated Framework for Implementation Research [31,32] to understand participants’ perceptions of app features, performance, personal opinions of the application, and suggestions for future improvement related to the study. Focus groups were conducted through Microsoft Teams, with both audio and video recorded, securely stored, and transcribed using Sonix software (Sonix Technologies). Deidentified transcripts were uploaded to a secure server and imported into NVivo software (version 13.1.7.1; Lumivero) for qualitative analysis. We had 1 participant who was unavailable to join the 3 scheduled focus groups. Instead, we interviewed this participant through Microsoft Teams after the 2-week trial.

### Data Analysis

Quantitative data were analyzed with descriptive statistics to summarize responses from the AIM, IAM, FIM, and uMARS. To explore potential variation in outcomes across participant characteristics, descriptive subgroup analyses were conducted by gender, primary mobility mode, injury level, and employment status. For each subgroup, mean (SD) values were calculated for all quantitative measures. Given the small sample size, no inferential statistics were computed, and all findings are reported as exploratory and descriptive only.

Transcripts were analyzed using an inductive-deductive hybrid thematic analysis approach, a qualitative analysis method commonly employed in mixed methods research [33]. To converge information from quantitative measures and qualitative focus groups, we first developed an initial codebook using uMARS domains as the high-level themes. Two primary coders, a project coordinator and a research trainee, conducted the qualitative analysis. Both had formal training in qualitative methods under the supervision of an experienced qualitative researcher and had prior experience in rehabilitation research. Neither coder had any personal or financial relationship with the app developer. To enhance analytic rigor and reduce potential bias, both coders independently reviewed and coded each transcript in NVivo, and they discussed and reconciled any discrepancies they encountered, refining the codebook as needed. The coders met regularly to review and harmonize their coding across multiple transcripts. When consensus could not be reached through discussion, a postdoctoral researcher with expertise in qualitative methods served as the final arbitrator. Through an iterative process, we refined the codes and organized them into themes and subthemes. Frequencies reported in the qualitative results reflect the number of coded references across all transcripts rather than the number of individual participants who mentioned each theme, consistent with classical content analysis frameworks for focus group data [34]. A single participant may therefore contribute more than one coded reference to a given theme.

In this process, we converged both quantitative and qualitative results by comparing the results of those measures with the codes and themes. This triangulation of findings helped us develop a richer, more nuanced understanding of participants’ user experiences with the app-based intervention.

### Ethical Considerations

The study was approved by the Institutional Review Board of Northwestern University (STU00216188). All participants provided written informed consent before enrollment. The study procedures adhered to the principles of the Declaration of Helsinki and institutional ethical guidelines. All data collected were deidentified before analysis to protect participant privacy and confidentiality. Participants received a US $100 honorarium upon completion of all study activities.

## Results

### Demographic Characteristics

**Figure 1.**
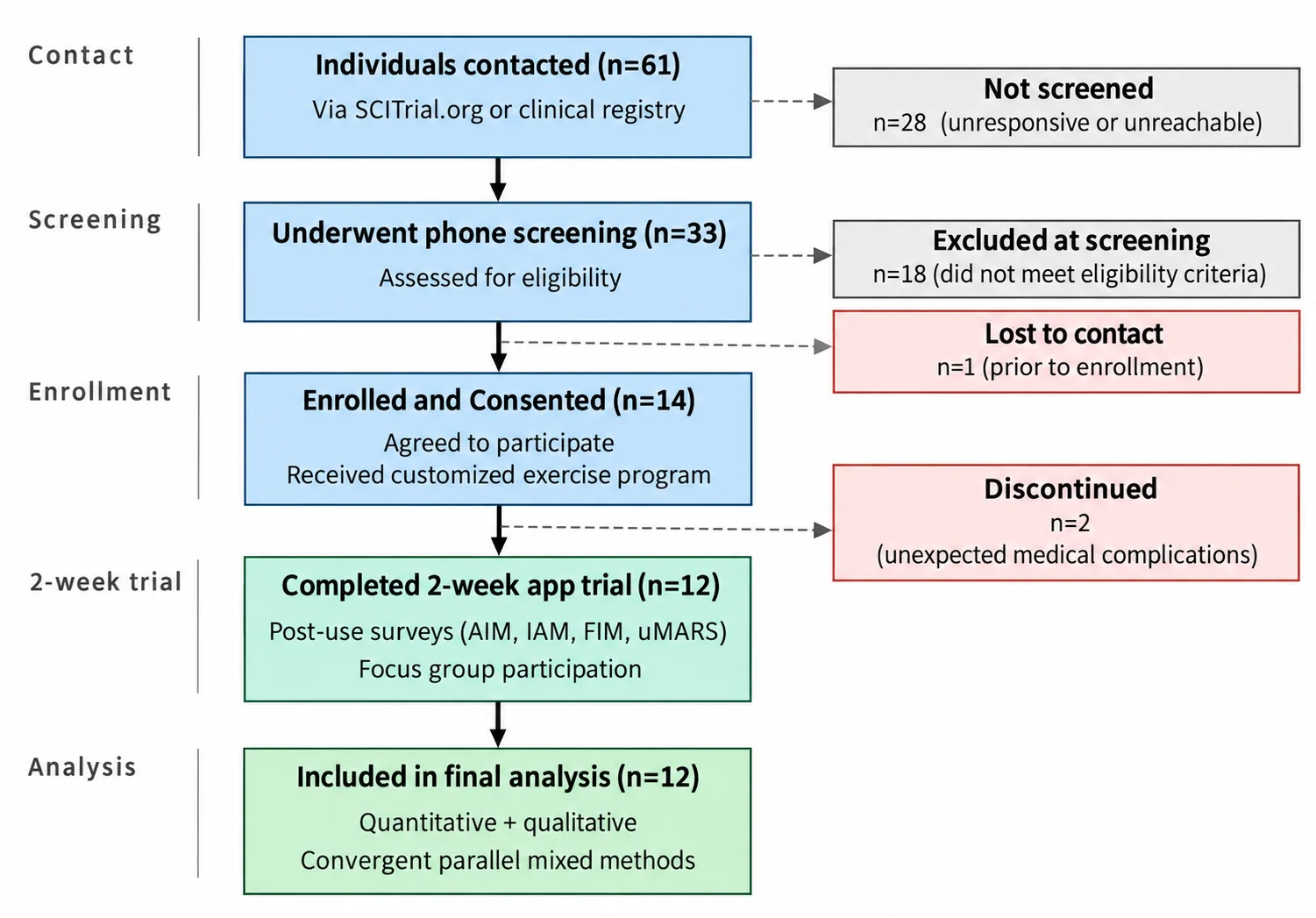
Participant flow diagram illustrating recruitment, screening, enrollment, and retention across study phases. AIM: Acceptability of Intervention Measure; FIM: Feasibility of Intervention Measure; IAM: Intervention Appropriateness Measure; SCI: spinal cord injury; uMARS: User Version of the Mobile Application Rating Scale.

Of the 61 participants initially recruited, 33 underwent phone screening for eligibility. Fourteen met the criteria and agreed to participate in the study. Of these, 86% (12/14) completed all study activities and were included in the final analysis (Figure 1), indicating good retention and supporting the protocol’s feasibility. Two participants were unable to complete the required activities due to unexpected medical complications. Participants’ demographic and clinical characteristics are presented in Table 1. The cohort had a mean age of 52 (SD 11) years and was evenly split by gender: 50% (6/12) male and 50% (6/12) female. Most participants identified as White individuals (8/12, 67%), and 50% (6/12) had a thoracic-level SCI.

**Table 1.** Demographic and clinical characteristics of 2-week trial participants.

Characteristic	SCI^a^ (N=12)
Age (y), mean (SD; range)	50.5 (17; 24-77)
Years postinjury, mean (SD; range)	14 (13; 1‐41)
Sex, n (%)	
Male	6 (50)
Female	6 (50)
Ethnicity/race, n (%)	
White	8 (67)
Asian	1 (8)
Multiracial	2 (16)
Unknown/not reported	1 (8)
Employment status, n (%)	
Full/part-time employed	3 (25)
Unemployed	2 (16)
Disability leave	3 (25)
Retired	4 (33)
SCI injury level, n (%)	
C1-C8	3 (25)
T1-T12	6 (50)
L1-L5	3 (25)
Type of paralysis, n (%)	
Incomplete	6 (50)
Complete	2 (17)
Unknown	4 (25)
AIS^b^ score, n (%)	
A	2 (17)
B	1 (8)
C	3 (25)
D	2 (17)
E	0 (0)
Unknown	4 (33)
Primary mobility, n (%)	
Walking (with or without an assistive device)	6 (50)
Manual wheelchair	3 (25)
Power wheelchair	1 (8)
Equally walk and use a wheelchair	2 (17)

a SCI: spinal cord injury.

b AIS: American Spinal Injury Association (ASIA) Impairment Scale.

### App Usage Patterns

App usage data provided objective behavioral indicators of feasibility and engagement during the 2-week trial. Seventy-five percent (9/12) of participants accessed the app at least once per week, with a median of 1.75 (IQR 0.88‐2.63) active days per week. Additionally, 58% (7/12) of participants left at least one in-app comment during the trial period, with a median of 3.5 (IQR 0‐8, range 0‐30) comments per participant. In-app comments included notes on exercise modifications, pain or discomfort during activities, and self-directed progressions (Multimedia Appendix 1).

### Quantitative Findings

**Figure 2.**
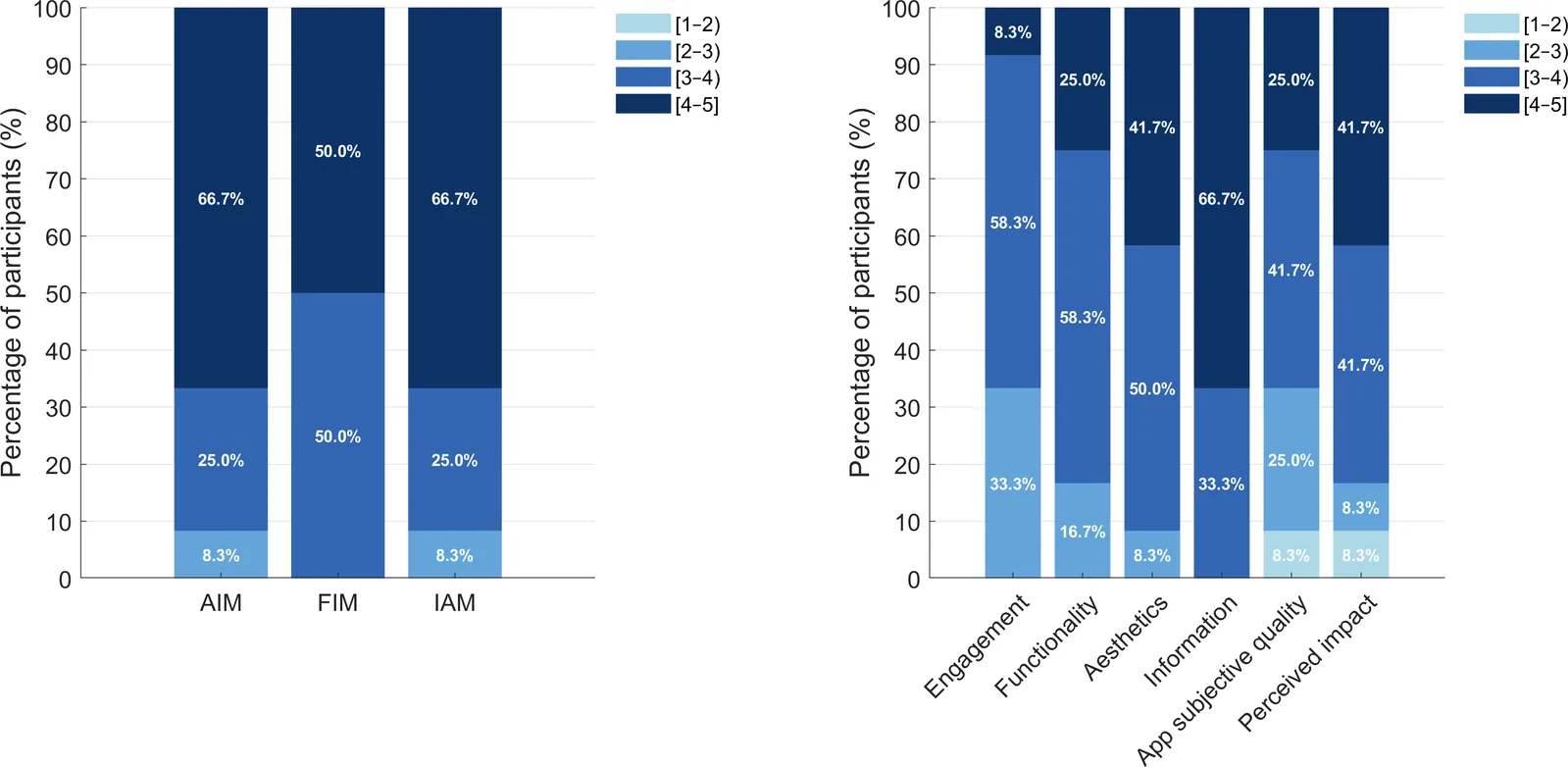
Distribution of participant ratings for implementation (AIM, IAM, and FIM) and app quality (uMARS) measures. Stacked bar plots display the distribution of participant responses to implementation measures (AIM, IAM, and FIM; left) and app quality ratings (uMARS; right). Each bar shows the proportion of participants whose mean score for a measure or subscale falls into the following ranges: 1‐2 (very low), 2‐3 (low), 3‐4 (moderate), and 4‐5 (high). Scores are based on Likert-scale items ranging from 1 (eg, “Completely disagree”/“Inadequate”) to 5 (eg, “Completely agree”/“Excellent”), and the resulting mean values represent aggregate ratings for each subscale or construct. AIM: Acceptability of Intervention Measure; FIM: Feasibility of Intervention Measure; IAM: Intervention Appropriateness Measure; uMARS: User Version of the Mobile Application Rating Scale.

Sixty-seven percent (8/12) of participants rated the program as highly acceptable and feasible (scores of 4 to 5), and 50% (6/12) rated it as highly appropriate (Figure 2, left panel). Among the 4 uMARS domains of objective quality, participants rated information most positively, with 67% (8/12) of responses in the high range (4-5; Figure 2, right panel). Aesthetics followed, with 42% (5/12) of responses in the high range (4-5). Functionality showed a broader distribution, with most ratings (7/12, 58%) in the moderate range (3-4), followed by 25% (3/12) in the top range (4-5) and 17% (2/12) in the low range (2-3). Engagement received the least favorable responses, with only 8% (1/12) of participants rating it in the high range (4-5), and 33% (4/12) rating it in the low range (2-3). Ratings for subjective quality and perceived impact were generally favorable. Eighty-three percent (10/12) of participants rated the perceived impact above 3, with 42% (5/12) rating it in the high range (4-5), and only 8% (1/12) rating it in the very low range (1-2). Similarly, most participants (8/12, 67%) rated the subjective quality above 3, while only 8% (1/12) rated it in the very low range (1-2). Table 2 shows the descriptive statistics (mean and SD) of the AIM, FIM, IAM, and uMARS scores.

**Table 2.** Results from the User Version of the Mobile Application Rating Scale and implementation outcome measures of acceptability, appropriateness, and feasibility.

Measures	Score, mean (SD)
Quality of mHealth^a^ app	
uMARS^b^—Objective app quality^c^	3.57 (0.52)
uMARS—Information	4.10 (0.52)
uMARS—Functionality	3.51 (0.66)
uMARS—Aesthetics	3.50 (0.70)
uMARS—Engagement	3.18 (0.72)
uMARS—Perceived impact	3.79 (0.92)
uMARS—Subjective app quality	3.27 (0.88)
Implementation measures	
Intervention Appropriateness Measure	3.85 (0.60)
Acceptability of Intervention Measure	3.83 (0.64)
Feasibility of Intervention Measure	3.81 (0.73)

a mHealth: mobile health.

b uMARS: User Version of the Mobile Application Rating Scale.

c Objective app quality was calculated as the average score of the information, functionality, aesthetics, and engagement subscales.

### Exploratory Subgroup Findings

Female participants tended to rate the app more favorably across all domains, with the largest differences observed in perceived impact (female: mean 4.22, SD 0.87; male: mean 3.36, SD 0.99) and AIM (female: mean 4.21, SD 0.40; male: mean 3.46, SD 0.64). Wheelchair users rated the app higher than walking-only users across most domains, especially for the FIM (wheelchair: mean 4.38, SD 0.48; walking: mean 3.38, SD 0.48) and aesthetics (wheelchair: mean 4.19, SD 0.48; walking: mean 3.08, SD 0.76). Lumbar-level participants rated information (mean 4.33, SD 0.38) and perceived impact (mean 4.17, SD 0.58) higher than those of the cervical and thoracic groups. Employment status showed minimal variation across most domains. These patterns should be examined in future studies using larger, more diverse samples.

### Qualitative Findings and Integration With Quantitative Findings

#### Integrated Findings

We coded a total of 292 quotations across 43 themes and subthemes (Table 3). We described them based on uMARS domains. Table 4 shows representative quotes from various themes and subthemes.

**Table 3.** Themes and subthemes identified from the focus groups^a^.

High-level theme	Mid-level theme	Lower-level theme	Theme frequency, n	Individual theme frequency, %	Theme definition
Engagement	—^b^	—	1	0.34	Participant mentions features that make the app fun, interesting, interactive, or customizable, including alerts, messages, reminders, feedback, or sharing options.
—	Entertainment	—	1	0.34	Participant mentions that the app includes fun or entertaining components.
—	—	Tracking progress	12	4.11	Participant mentions the importance of tracking workout history and visualizing progress over time.
—	—	Virtual human guide	3	1.03	Participant mentions interest in having a virtual trainer to demonstrate movements, provide motivation, or guide workouts.
—	—	Rewards	4	1.37	Participant mentions intrinsic or extrinsic rewards as motivating factors for using the app.
—	Interest	—	0	0.00	Participant mentions whether the app is engaging or presents information in an interesting way compared to other apps.
—	—	Variety	3	1.03	Participant mentions appreciation for a diverse range of exercise options.
—	Customization	—	2	0.68	Participant mentions the ability to personalize app settings such as sound, content, or notifications.
—	—	Customize schedule	15	5.14	Participant mentions features that allow scheduling and tracking of completed or pending exercises.
—	—	Customize exercise	19	6.51	Participant mentions the ability to tailor or modify exercises to better suit personal needs, abilities, or routines.
—	Interactivity	—	4	1.37	Participant mentions features that allow user input, deliver feedback, or provide prompts such as reminders or sharing options.
—	—	Notifications and reminders	12	4.11	Participant mentions the usefulness and timing of alerts to stay on track or suggests improvements to notification delivery.
—	—	Feedback	3	1.03	Participant mentions writing reflections or thoughts about their experiences in the app.
—	—	Postexercise surveys	5	1.71	Participant mentions experiences with postexercise feedback surveys, including their appearance, timing, and relevance.
—	—	EMA^c^	12	4.11	Participant mentions experiences with EMA surveys, including preferences for timing, frequency, and simplicity.
—	—	Connection with others	3	1.03	Participant mentions features for interacting with other users, such as chat spaces or community options.
—	Target group	—	1	0.34	Participant mentions whether the app content (visuals, language, and design) is appropriate for the target audience.
—	—	Patients with SCI^d^	7	2.40	Participant mentions the relevance of the app’s content and features for individuals with SCI, including condition-specific references and exercises.
Functionality	—	—	1	0.34	Participant mentions the app’s technical performance, ease of use, logical navigation, and overall design, including its intuitive operation.
—	Performance	—	27	9.25	Participant mentions how accurately and quickly the app’s features and components (eg, buttons and menus) function.
—	Ease of use	—	9	3.08	Participant mentions how easy it is to learn to use the app and how clear the instructions, icons, and menu labels are.
—	Navigation	—	3	1.03	Participant mentions whether navigation between screens feels logical and whether all necessary links and pathways are present.
Aesthetics	—	—	—	—	Participant mentions the overall visual design of the app, including graphic quality, color scheme, and stylistic consistency.
—	Layout	—	3	1.03	Participant mentions whether the arrangement and size of buttons, icons, menus, and on-screen content are appropriate.
—	Graphics	—	3	1.03	Participant mentions the quality and resolution of visual elements such as buttons, icons, and menus.
—	Visual appeal	—	6	2.05	Participant mentions how visually appealing the app is overall.
Information	—	—	—	—	Participant mentions the presence of high-quality, credible information in the app, including text, feedback, assessments, or references.
—	Quality of information	—	5	1.71	Participant mentions whether the app content is accurate, well-written, and relevant to its intended goals.
—	—	Exercise content	17	5.82	Participant mentions various formats of exercise instruction in the app, including written steps, pictures, and videos.
—	Visual information	—	—	—	Participant mentions whether visual explanations, such as graphs, images, or videos, are clear, accurate, and easy to understand.
—	—	Design quality	23	7.88	Participant mentions how clearly the app’s information is laid out and how easy it is to navigate or comprehend.
—	—	Weekly insights	5	1.71	Participant mentions the usefulness and clarity of the feature summarizing weekly activity.
Perceived impact	—	—	—	—	Participant mentions how the app influenced their thoughts, feelings, or behaviors related to the targeted health behavior.
—	Awareness	—	1	0.34	Participant mentions that the app increased their awareness of the importance of the targeted health behavior.
—	—	Participant exercise capability	5	1.71	Participant mentions their physical limitations, fluctuating abilities, and emotional responses when completing exercises.
—	Knowledge	—	4	1.37	Participant mentions that the app improved their understanding or knowledge of the health behavior.
—	Intention to change	—	5	1.71	Participant mentions that the app increased their motivation or intention to change their behavior.
—	—	Accountability/motivation	14	4.79	Participant mentions that the app helped them feel more responsible and motivated to stay engaged with the program.
—	Attitudes	—	26	8.90	Participant mentions that the app positively influenced their attitudes toward improving their health behavior.
—	—	Compatible with routine	12	4.11	Participant mentions how well the app fits into their daily schedule and time constraints.
—	Help seeking	—	—	—	Participant mentions that the app encouraged them to seek further help or professional support if needed.
—	—	Clinician assistance	11	3.77	Participant mentions the role or usefulness of clinician interaction or assistance in conjunction with the app.
—	Behavior change	—	5	1.71	Participant mentions that using the app led to increases or decreases in the targeted health behavior.
Total references	—	—	292	—	—

a Themes and subthemes were identified from focus group data, based on the user version of the Mobile App Rating Scale categories. Theme frequencies reflect the number and percentage of individual comments coded within each category.

b Not applicable.

c EMA: ecological momentary assessment.

d SCI: spinal cord injury.

**Table 4.** Representative participants’ quotations by themes^a^.

High-level theme	Representative quotations
Engagement	“That’s always very reassuring to people because you sometimes don’t make a lot of progress quickly, and you start getting a little disappointed in yourself. Then if you see that, 'Oh, well, this was just a bad week, because when I started this, I could only do two of these and now I can do 14.' That type of a visual would be very, very helpful.” [q1; *Tracking progress*; Positive] “I like to be able to do it based on my schedule because some mornings I may not have enough energy to do all of them. I just do a couple, and later in the day, I can do the rest.” [q2; *Customized schedule*; Positive] “Get rid of the jumping jacks and substitute something else. I felt silly doing them.” [q3; *Customized exercise*; Mixed] “I liked having the exercises tailored to me, initially by the physical therapist, based on what I needed to work on the most, so that was helpful.” [q4; *Customized Exercise*; Positive] “I kind of need that reminder because my day gets away from me every day.” [q5; *Notifications and reminders*; Positive] “Maybe if you sent out four reminders a day, like, 'Hey, did you do your exercise yet?' That would help me stay on track.” [q6; *Notifications and Reminders*; Positive] “If you can’t finish, being able to provide that easily and provide that feedback is important. Otherwise, the therapist who’s going to look at your data is not going to know the reason you didn’t finish.” [q7; *Post Exercise Surveys*; Positive] “The surveys helped give you guys a better understanding of how we, the users, used it after our exercise.” [q8; *Ecological Momentary Assessment (EMA*); Positive] “I didn’t get any of the surveys, which was disappointing.” [q9; *Ecological Momentary Assessment (EMA*); Negative]
Functionality	“My notifications never really worked. I guess I had it correctly set up in my phone, but I never got notifications like when to start or that they were due.” [q10; *Performance*; Negative] “I didn’t understand the exercise lingo at first, but the step-by-step guide was helpful.” [q11; *Ease of use*; Mixed] “A video of how to use the app, especially when starting off, would be helpful [...]. They can always go back and watch the video again if needed.” [q12; *Ease of use*; Positive] “It would be much more helpful if you saw the first thing for an exercise, and there was one action per screen.” [q13; *Navigation*; Negative]
Aesthetics	“Once you get there, it starts out by just showing you all these different options, just very visually oriented, not just the exercises themselves.” [q14; *Visual Appeal*; Positive] “Having a trend chart like we just mentioned. That’s a key one. If you showed it as a pie chart. Visual things are more helpful.” [q15; *Graphics*; Mixed]
Information	“I like the way they were described. Kind of with ’One, two, three, four,' the steps to do it.” [q16; *Exercise Content*; Positive] “I used both. I used the video too.” [q17; *Exercise Content*; Positive] “Because it’s associated with the [Institution Name], your expectations are very high because the quality of the institution is so tremendous.” [q18; *Quality of information*; Positive] “Seeing how I improved week to week was very reassuring.” [q19; *Weekly Insights*; Positive]
Perceived impact	“I think the intent is good. What’s trying to be done is a good idea.” [q20; *Attitude*; Mixed] “I really wanted to love this program, but I just couldn’t fall in love with it. It just really needs to be fine-tuned much more overall.” [q21; *Attitude*; Negative] “It makes me more motivated. Give me a chance just to start my whole day moving rather than lying around or doing nothing originally and then waiting to go to the gym throughout the day.” [q22; *Accountability/Motivation*; Positive] “It was compatible because it just gave us users a reminder to be active for exercise. So yeah, it was like good timing, I think in my opinion, gave us something to do uh, throughout the day.” [q23; *Compatible with routine*; Positive] “That’s why I want this app to work more because I like that we’re getting stuff from actual PTs instead of somebody who doesn’t really know how the spinal cord works and different things like that. And I love it.” [q24; *Clinician assistance*; Positive]

a Quotations are labeled with (qX*; Lower/Mid-level theme; Valence [Positive/Negative/Mixed]*) to indicate the original identifier and thematic categorization.

#### Engagement

Participants viewed tracking progress over time as a valued feature, noting that visualizing improvement could boost motivation and offer reassurance during tough times (q1; *Tracking progress*).

They highlighted the importance of customizing the app, especially adapting the exercise programs and schedules to their daily energy levels and availability (q2; *Customized schedule*).

Additionally, participants appreciated the option of modifying exercises to match their abilities and preferences, with some recommending the removal or substitution of exercises they found unsuitable (q3; *Customized exercise*). Participants especially appreciated the program’s flexibility, including how it customized exercises to meet individual needs (q4; *Customized exercise*).

Participants favored regular modifications, such as weekly or biweekly updates, to stay engaged. Participants discussed various aspects of interactivity within the app. Notifications and reminders were often noted as essential tools for maintaining participants’ engagement with the program (q5 and q6; *Notifications and reminders*).

Feedback, including postexercise surveys, was another key focus. Participants appreciated the opportunity to provide detailed explanations when they were unable to complete an exercise (q7; *Postexercise surveys*).

The daily EMA surveys received mixed feedback. While some participants found them helpful for capturing real-time experiences and monitoring progress throughout the day, most comments expressed frustration with technical issues, such as surveys disappearing before they could finish (q8 and q9: *EMA*).

#### Functionality

Participants shared diverse feedback on the app’s functionality, emphasizing the need for improved performance, easier use, and smooth navigation. Participants expressed frustration that the app’s features did not function as expected, and some noted inconsistent or unreliable notifications, which reduced their overall experience (q10; *Performance*). All these comments were pertinent only for the current study.

Participants emphasized ease of use as a key factor. While many comments reflected appreciation for the detailed instructions and step-by-step exercise guides, some highlighted difficulties with exercise terminology. Navigation challenges were also a recurring theme, with some participants requesting more accessible resources, such as tutorial videos (q11 and q12; *Ease of use*).

Participants suggested that simplifying the app’s interface by reducing the number of actions per screen could greatly improve usability (q13; *Navigation*).

#### Aesthetics

Aesthetic appeal was discussed in both the quantitative and qualitative results. The qualitative findings revealed mostly positive to neutral opinions, with notable mentions of the appreciation for and helpfulness of the visuals (q14; *Visual appeal*).

Other participants provided suggestions on how to make certain information more visually appealing (q15; *Graphics*).

#### Information

Qualitative feedback highlighted participants’ appreciation for the structured instructions and the option to choose between written and video guides, highlighting the importance of multimodal resources (q16 and q17; *Exercise content*).

The credibility of the app’s content was also viewed positively, with participants noting that having exercises and materials developed by clinicians from a reputable research institution increased their confidence in the quality and reliability of the content (q18; *Quality of information*).

Features such as visual layouts and progress tracking were praised for their motivational value. These insights not only explain the high score in the Information domain but also highlight the app’s ability to effectively provide tailored, accessible, and motivating content to support individuals with SCI in adhering to their exercise programs (q19; *Weekly insights*).

#### Perceived Impact

Qualitative feedback on this domain was more nuanced than the generally positive quantitative ratings. Participants reported room for improvement, suggesting further refinements (q20 and q21; *Attitudes*).

Some comments noted that the app could positively influence participants’ motivation to increase and improve physical activity (q22; *Accountability/Motivation*). Many others highlighted that the app helped them stay accountable by fitting into their daily routines, which increased the effectiveness and accessibility of exercise in their lives (q23; *Compatible with routine*).

Although some participants expressed apprehension about using the app, most comments were positive about the model of clinician-supported app use. The involvement of SCI-trained physical therapists was identified as a key facilitator of trust and engagement (q24; *Clinician Assistance*).

Overall, participants agreed that the app has the potential to improve exercise adherence within the SCI community after certain modifications by boosting motivation, accountability, and incorporating clinical expertise.

## Discussion

### Interpretations and Implications of Findings

This study aimed to evaluate the acceptability, appropriateness, feasibility, and quality of a customized mHealth app designed to support home-based exercise participation among individuals with SCI. Using a mixed methods approach that converged quantitative survey data and qualitative feedback from focus groups, we gained a comprehensive understanding of users’ experiences, perceptions, and recommendations for improving the app specific to the study’s needs.

The intervention was generally viewed as acceptable, appropriate, and feasible, as reflected in both the survey responses and participant narratives. Among the uMARS domains, the information domain emerged as the highest-rated component and a key strength of the intervention. Users appreciated the app’s structured content, the relevance of the prescribed exercises, and the clear instructions provided through written guidance and demonstration videos. Participants valued the credibility of the app’s content because it was developed with input from SCI-specialized physical therapists and affiliated with a reputable rehabilitation institution, elements known to increase user trust and engagement in digital interventions [35,36]. These results align with the findings of Bernard et al [20] and Hayat et al [37], which noted that multimodal content can greatly enhance accessibility and comprehension for users with diverse functional needs.

Engagement varied across users. App usage data confirmed that the majority of participants accessed the app regularly and left in-app comments throughout the trial, indicating a meaningful level of interaction with the platform. These comments documented exercise modifications, physical responses, and self-directed progressions, providing the clinical team with ecologically valid, real-world insights into each participant’s exercise performance, information that can directly inform ongoing program refinement and personalization. However, the level of engagement varied considerably across individuals, potentially reflecting differences in motivation, physical capacity, or technology familiarity. Qualitatively, while some participants appreciated the flexibility of scheduling and progress tracking, others reported limited interaction and support for maintaining motivation. Technical challenges with reminders and EMA delivery, along with the absence of interactive feedback or adaptive goals, were seen as barriers to deeper engagement. Notably, issues with EMA delivery, in which some participants did not consistently receive daily surveys as intended, likely reduced opportunities for real-time interaction with the app and may have contributed to the comparatively lower ratings observed in the Engagement domain of the uMARS and the FIM. These findings should therefore be interpreted in light of this technical limitation. Participants expressed interest in enhancements such as motivational messages, reward-based features, and more personalized reminders. These observations align with the broader mHealth literature emphasizing that personalization and adaptive feedback are critical for sustained engagement in populations with chronic conditions or mobility limitations [25,38]. Similar findings have been reported in tele-exercise programs for populations such as individuals with stroke and multiple sclerosis, where user-centered, adaptive features were key to feasibility [39,40].

Feedback on the Functionality was mixed. While participants appreciated the step-by-step exercise instructions, they noted inconsistencies in navigation, difficulties with exercise terminology, and a need for onboarding tools, such as tutorial videos. These findings echo those of Haley et al [35] and Brown et al [41], emphasizing that ease of learning, early-stage guidance, and intuitive design are essential for successful mHealth adoption, particularly among users with varied levels of digital literacy.

Aesthetic design was generally viewed favorably, with users appreciating the layout and visual clarity. Suggestions for improvement focused on progress graphics and charts, consistent with prior evidence that visual analytics and feedback tools enhance motivation and reinforce behavior change in home-based rehabilitation programs [42]. These suggestions highlight that effective visual design focuses not just on appearance but also on promoting behavior change.

The perceived impact was positive among participants. Many described the app as helpful in increasing accountability and supporting the integration of exercise into their daily routines. The association with trained clinicians was highlighted as a factor that boosted participants’ confidence and the perceived value of the program. These findings are consistent with studies by van Eetvelde et al [43] and Nataletti et al [25] emphasizing the importance of tailored, clinically credible digital tools in supporting behavior change and self-management in rehabilitation populations. The higher ratings for perceived impact than for engagement reflect an important conceptual distinction. Perceived impact reflects participants’ beliefs about the intervention’s potential to improve their health behavior, shaped primarily by the quality and personalization of the app’s content and by the credibility of clinician involvement. By contrast, engagement depends on specific interactive features, such as notifications, real-time feedback, and adaptive goal setting, that sustain active app use over time. Participants may therefore recognize the value of an intervention while still facing barriers when these features underperform, including the EMA delivery issues documented in this study.

Exploratory subgroup analyses revealed preliminary patterns that warrant further investigation. Female participants and wheelchair users tended to rate the app more favorably across most domains, particularly in adoption intent and perceived impact, suggesting the app may resonate differently across gender and mobility profiles. Lumbar-level participants rated information quality and perceived impact slightly higher than those in other injury groups, while employment status showed minimal variation across domains. These patterns are preliminary, given the small and unequal subgroup sizes, and should be interpreted with caution. They highlight the importance of examining subgroup differences in future studies using larger, more diverse samples.

### Limitations and Future Directions

This study has several limitations. The small sample size, geographically limited recruitment, and missing neurological classification data may constrain sample characterization and reduce generalizability. The 2-week exposure period precludes conclusions about long-term adherence or sustained engagement with the app; these outcomes will be assessed in the subsequent efficacy study. Additionally, technical issues with EMA delivery prevented some participants from receiving the daily surveys as intended, potentially limiting engagement with the app and affecting the feasibility ratings.

The findings of this study will directly inform the refinement of specific app features by the app developer prior to a subsequent proof-of-concept efficacy study. Development priorities include resolving EMA delivery issues, enhancing notification and reminder features, introducing reward-based engagement mechanisms, and simplifying navigation. Future research should evaluate the effectiveness of the refined mobile app-based exercise intervention in promoting adherence to SCI-specific exercise guidelines and improving health outcomes among a larger, more diverse sample of community-dwelling individuals with SCI over a multimonth intervention period to better capture sustained engagement and long-term outcomes. Objective usage metrics and subgroup analyses across demographic and clinical characteristics should be expanded to better understand engagement patterns and identify who benefits most from the intervention. More broadly, this study underscores the value of centering end-user feedback in the early design stages to develop mHealth tools that reduce barriers to access, promote behavior change, and improve quality of life for populations with mobility needs.

### Conclusion

This study highlights the value of a user-centered, mixed methods approach for evaluating an mHealth intervention in individuals with SCI. The intervention was perceived as informative, credible, and accessible and closely aligned with users’ physical activity needs. Meanwhile, participants recognized opportunities to enhance functionality and engagement based on their experience using the intervention as individuals with SCI. As a next step, this user feedback can guide further refinement of relevant app features by the app developers to better meet the usability and accessibility needs of individuals with SCI. Subsequent evaluation through a field test is necessary to determine if the intervention can increase adherence to SCI-specific physical activity guidelines and ultimately improve users’ health and quality of life. These findings align with the wider digital health literature and highlight the importance of customizing design and content to meet user needs. By incorporating these refinements, mHealth interventions can more effectively support exercise adherence, reduce barriers to rehabilitation, and support long-term health in the SCI population.

## Supplementary material

10.2196/84316 Multimedia Appendix 1 Representative in-app comments by participant.

## Abbreviations

AIM: Acceptability of Intervention Measure
EMA: ecological momentary assessment
FIM: Feasibility of Intervention Measure
IAM: Intervention Appropriateness Measure
mHealth: mobile health
SCI: spinal cord injury
uMARS: User Version of the Mobile Application Rating Scale
