# Supplementary material for: Exploring the Usability and Quality of a Mobile App–Based Intervention for Personalized Exercises in Individuals With Spinal Cord Injury: Convergent Parallel Mixed Methods Study
Source: JMIR Form Res. 2026 Sep 25;10:e84316. doi: 10.2196/84316 (PMC13614398; doi:10.2196/84316)
Supplement: Supplementary file 1 [file formative-v10-e84316-s001.docx]

# Multimedia Appendix 1

## Representative In-App Comments by Participant

The following table presents a selection of representative in-app comments left by participants during the two-week trial. Comments reflect real-world exercise performance including self-reported modifications, physical responses, and self-directed progressions. All participants are identified by anonymized codes (P01-P06). Comments have been lightly edited for readability where necessary (e.g., spelling) but are otherwise reproduced verbatim. Comments from one participant are not included in this table as they contained sensitive personal health information.

| **Participant** | **In-app comment** |
| --- | --- |
| P01 | 30 minutes bike |
| P01 | Timer won’t work. |
| P01 | Done on Wednesday. 15 minutes. |
| P02 | My right ankle is a little stiff, so a little harder on the right ankle but it felt good |
| P02 | It’s hard to control my hips to keep my knees from falling to the side sometimes |
| P02 | I did it without the timer |
| P02 | I clicked on the wrong day. I have been walking every day but doing the program on Tuesdays and Thursdays. |
| P02 | Very easy |
| P02 | I must’ve swiped away from the timer — it did not calculate 20 minutes. It was so nice outside. |
| P02 | Kept getting a charley horse |
| P03 | 1 set done |
| P03 | Can barely lift my left foot |
| P03 | 40 minutes on some uneven terrain |
| P04 | Hard to balance |
| P04 | Didn’t use weight, will use next time |
| P04 | I did it laying down today to get a better stretch instead of struggling to balance |
| P04 | Didn’t finish, got derailed by phone |
| P04 | Shoulder pain, didn’t finish |
| P05 | I especially felt the stretch on my left side. |
| P05 | Used 3 lb weights. Hand strap to secure dumbbell to left hand. |
| P05 | Went for a walk outside this evening for about 20 min. Using cane. |
| P05 | Started to rain — rode for about 20 min |
| P06 | Swam and stretched |

*Note: Participants who did not leave any in-app comments during the trial period are not represented in this table.*
